# Collective Research for General Practitioners

**Published:** 1913-11-22

**Authors:** 


### COLLECTIVE RESEARCH FOR GENERAL PRACTITIONERS.

A circular has been widely distributed just lately to members of the medical profession inviting them to join a proposed "Association of General Practitioners for Collective Research." The proposal may or may not "catch on" amongst those to whom it is addressed, but the idea, as unfolded in the circular, is distinctly ingenious, and deserves a brief examination. The general practitioner sees a very different picture of disease from that which presents itself to the specialist and the hospital consultant; and this fact accounts for certain divergencies of method and treatment which the specialist is inclined to label pig-headedness or ignorance on the part of the practitioner, and the latter often calls unpractical faddism on the part of the former.

As an example of these tendencies, and to show that each side has a certain amount of justification on occasions, the cases of blood counts and of appendicitis will suffice, though hundreds more could be mentioned of the same type. A great many specialists attributed, a few years ago, such importance to the enumeration of the white corpuscles of the blood that they considered no case completely investigated until this had been done, and they allowed enormous weight to the result in settling their diagnosis and treatment. The general practitioner sniffed at this innovation as too laborious and expensive to be practical, and too fallacious to be of any use, and the general practitioner was right, as even the mass of consultants eventually had to admit. With regard to appendicitis the boot was rather on the other leg. The operating surgeon found himself confronted with so many serious cases of neglected appendicitis, in which the death-rate, despite his best endeavours, was exceedingly high, that he pleaded for immediate operation in all cases as soon as the diagnosis should be established. The general practitioner, on the other hand, saw so many mild cases recovering without operation that he was inclined to reject the doctrine of instant operation as exaggerated. In time, however, experience of the unpredictable vagaries of the disease and of the very favourable results of early operation compelled the general practitioner to come round to the specialist's view.

The fact remains, nevertheless, that general practitioners and consultants do get very different view points of many diseases, and that clinical researches conducted solely by the latter class are necessarily in some degree one-sided. In general terms it is true that general practitioners do not carry out research of any great value, partly because their time is too much occupied by the necessity of earning a living, partly because they do not as a rule come across large numbers of cases of the same disease. There have been, of course, brilliant exceptions to this rule whom it is not necessary to specify, but it holds good for the bulk. On the other hand, it is *par excellence* the general practitioner who sees the beginnings of disease, when early and often obscure symptoms have to be interpreted; there is much sense in the old saw, "When any fool can make the diagnosis, the wisest cannot cure the patient." The practitioner, too, sees the after-results of new forms of treatment, especially of operative treatment, and is much more closely concerned with the last stages of incurable disease than the specialist. "He is the best judge," says the circular above mentioned, "of the practical value of research." His great limitation, the small number of cases of each disease which he is likely to encounter, can be obviated, it is thought, by adopting the principle of collective research.

The circular propounds twelve subjects for research for the ensuing three months. Practitioners who join the Association are invited at the end of the quarter to send in their observations on all cases which fall within the prescribed limits and bear upon the queries asked concerning the twelve subjects.

The first thought that will occur to the critical or the censorious will be a doubt of the motives of the promoters of the scheme. It is easy to see that it might be made the stalking-horse for obnoxious and unethical methods of "pushing business," if those who are getting it up are of the self-advertising fraternity ridiculed lately in our leading medical journal under the felicitous heading of "The Boundary Line." On the face of it, we see no solid ground for such apprehensions in the present instance, but the unknown men whose names are appended to the circular will have to conquer this suspicion and live it down if their Association is to make headway. Knowing nothing either for or against them, we fall back on the internal evidence of the circular, and are able to say that it betrays nothing which can offend.

We are disinclined to throw cold water on a scheme which is quite probably well intentioned and essentially honest. But we doubt whether collective research of this kind is really worth the trouble, and whether its results will be as reliable and valuable as the promoters hope. In an Association of this kind everything depends on the energy and ability of the men who are the mainsprings of the action. A provisional committee is mentioned, but no names are given. The chairman is a very young Cambridge graduate, who has had a good experience in resident posts at the London Hospital. The secretary is another Cambridge and London Hospital man, slightly senior to his chairman. If these gentlemen possess intellects as acute and energy as great as the late Sir Jonathan Hutchinson, together with abundant leisure, tireless enthusiasm, and the real scientific spirit of search after truth, the Association may accomplish great deeds. But unless those conditions are fulfilled we doubt whether much will be achieved, though we are willing to give the two London Hospital men all credit for good intentions. The list of subjects for research shows ability and knowledge of everyday practice, and, lastly, it should be added that no subscriptions are asked for.

